# The Increasing Sterility of American Women*Read at Gynecological Section American Medical Association, St. Paul, June, 1901.

**Published:** 1901-09

**Authors:** George J. Englemann

**Affiliations:** Boston


For Texas Medical Journal.

### The Increasing Sterility of American Women.*

BY GEORGE J. ENGLEMANN, M. D., BOSTON.

This investigation is based upon numbers which may seem small to admit of deductions as to conditions existing throughout a great country, but I feel justified in doing so, as the data are exact and cases are carefully sifted, in addition, many details are corroborated to a decimal by independent observers in far distant points: by Dr. Wilbur, in the census of Michigan, and Drs. Abbott and Kuszynsky in that of Massachusetts, by the careful observations of Dr. Chadwick in Boston and for the 18th century, by town records from Massachusetts communities. Certain data are taken from each, as no one investigation covers all the points I have developed, and some have never before been presented, so that no record for comparison exists: all are indirectly corroborated by correlated facts. Whatever may be thought of the results obtained, the data presented certainly suffice to indicate the imperative need for further and more extended investigation in this direction.

The sterility of woman has increased hand in hand with the much discussed decrease of fecundity, everywhere to some extent, but in the United States to an excessive degree, as fecundity has diminished more rapidly in other countries: from a sterility of two per cent in the eighteenth century, and a fecundity of five children to the marriage, conditions better than in any other country, and such

---

*Read at Gynecological Section American Medical Association, St. Paul, June, 1901.

as led to the Malthusian theory of super-fecundation, to the fear of overpopulating the earth's surface, after a lapse of one century, from first we have passed to last, and the other extreme is now presented: sterility greater and fecundity less than that of the women of any other nation, unless it be of France, who for this reason must yield her proud position of one-time supremacy and retrograde to the rank of a second-class power.

Among the laboring class in St. Louis 21 per cent. of all marriages are sterile, 24 per cent. among the higher classes, of foreigners only 17 per cent.; throughout the State of Massachusetts, Americans 20.2 per cent., foreigners 13.3 per cent., and in the city of Boston nearly 25 per cent. Among the laboring class, American born, the fecundity in the eighteenth century was five children to all marriages, at the beginning of the nineteenth century 4.5, is now and was at the end of that century 1.8 to 2.1; 2.1 in Missouri, 1.8 in Michigan, 1.8 in Boston, somewhat more among American born of foreign parentage, much more among .foreigners, among the Irish 4.2 in St. Louis, 3.5 in Boston, 5 in Michigan; among Germans 3.4 in St. Louis, 6 in Michigan, and in Massachusetts for all foreigners 4.9 children to the marriage.

Fecundity is somewhat less among the native American, also among the higher classes, least of all among college graduates, 1.6 childern to the married couple, in England 1.5, whilst for the population at large it is 4.2.

I have called attention to the frequency of miscarriage and divorce as concomittants and causes of sterility, mainly to emphasize that barrenness is not altogether due to physical causes, to pelvic disease amenable to local treatment, and that sterility is but too often artificially produced, the result of moral causes or the sequence to intentional miscarriage and the methods which precede it, the prevention of conception, both of which competent investigators have shown to be far too frequent. Divorce in Canada, one to 63,000 marriages; in England, one to 11,600; in Germany, one to 13,000; in France, one to 12,500; in all United States, one to 185; Massachusetts, one to 18.8; Rhode Island, one to 8.2 marriages.

Miscarriages are found in the proportion of one to 2.8 labors at term among Americans; one to 5.5 is the usually accepted standard. Among Americans of American parentage the frequency is somewhat greater, one to 2.7; among Americans born of foreign parentage somewhat less, both in St, Louis and Boston, one to three; among negroes, worse.

There is an absolute and primary barrenness, due to utero-ovarian disease, mainly to atresia, gonorrhea and to endometritis, with acrid discharge destructive to the spermatozoa; this is here for the first time distinguished from relative or secondary sterility, i. e., conception and miscarriage. This primary sterility is much less frequent, twelve per cent. among Americans, ten to twelve per cent among foreigners, which of course means relative sterility for Americans nine to twelve per cent., for foreigners three to six per cent., showing that among American born there is a much greater proportion of sterility, of childlessness due to abortion; this may be due to disease or traumatism accidental, more often authorities say not: much of the barrenness of womau is intentional. All sterility was in the American two per cent, in parts of Russia it is today 2.8 per cent., in Norway 2.5 per cent.; hence primary barrenness certainly in this country can not be over eight per cent.; my records show nine per cent., eight per cent. of twenty to twenty-three per cent of the childless. Even in absolutely primarily barren marriages sterility is once in four or five cases due to the male, showing that absolute sterility in a woman is not common, and that sterility is not mainly due to utero-ovarian disease is evident from its rapid increase, hand in hand with the astounding progress of gynecological science, which we have every reason to believe would reduce the number of childless women to a minimum were sterility referable to tangible physical causes.

Sterility is a sad affliction for the innocent sufferer, and for her our best efforts must be exerted; but if so rarely due to pelvic malformation and disease, why do I present these thoughts to the gynecological section of a medical society? It is because we must seek to stay the progress of this abnormal state, because men and women are in ignorance of the suffering prone to follow wilful and self-inflicted sterility; and it is this subject which claims a prominent chapter in the gynecology of the future—in preventative gynecology.

The death rate of nations has steadily decreased in the last decade by the development of preventive medicine, and so may sterility decrease and birth rate increase with the progress of preventive gynecology.

**INCREASE OF STERILITY AND THE CORRELATED FACTORS, FECUNDITY, MISCARRIAGE AND DIVORCE, IN THE UNITED STATES, 1800-1900.**

| | Generally accepted average. | Most favorable conditions *now* existing. | American colonies in the 18th Century. | United States at present and at close of 19th Century. |
|---|---|---|---|---|
| STERILITY.............. Per cent. of marriages childless. | 11. per ct. | 2.5 per ct. | 2. per ct. | 21.-23. per ct. General Average. 33. per ct. Col ege Graduates. |
| FECUNDITY ............ No. of children to each marriage. | 4.5 per ct. | 9.0 per ct. French Canadian. 7.2 per ct. Kaluga District, Russia. 6.4 per ct. Christiana. | 4.5-6. per ct. | 1.8 per ct. General Average. 1.4 per ct. College Graduates United States and England. |
| MISCARRIAGE......... No. of full-term labors to one abortion. | 5.5 per ct. | 3.3-5.5 per ct. | ..... ........ | 2.8 per ct. |
| DIVORCE .............. No. of marriage to one divorce. | ......... ..... | 63,000 Canada. 11,000 England. | .............. | 185. per ct. United States, 1890 18.7 per ct. Massachusetts 8.2 per ct. Rhode Island. |

